# *ERBB4* Promoter Polymorphism Is Associated with Poor Distant Disease-Free Survival in High-Risk Early Breast Cancer

**DOI:** 10.1371/journal.pone.0102388

**Published:** 2014-07-18

**Authors:** Kari J. Kurppa, Matjaz Rokavec, Maria Sundvall, Pirkko-Liisa Kellokumpu-Lehtinen, Heikki Joensuu, Hiltrud Brauch, Klaus Elenius

**Affiliations:** 1 Department of Medical Biochemistry and Genetics and Medicity Research Laboratories, University of Turku, Turku, Finland; 2 Turku Doctoral Programme of Molecular Medicine, Turku, Finland; 3 Dr. Margarete Fischer-Bosch-Institute of Clinical Pharmacology, Stuttgart, Germany; 4 University of Tübingen, Tübingen, Germany; 5 Department of Oncology, Turku University Hospital, Turku, Finland; 6 Department of Oncology, Tampere University Hospital, Tampere, Finland; 7 Department of Oncology, Helsinki University Central Hospital, Helsinki, Finland; CNR, Italy

## Abstract

A number of genetic variants have been linked to increased risk of breast cancer. Little is, however, known about the prognostic significance of hereditary factors. Here, we investigated the frequency and prognostic significance of two *ERBB4* promoter region variants, −782G>T (rs62626348) and −815A>T (rs62626347), in a cohort of 1010 breast cancer patients. The frequency of nine previously described somatic *ERBB4* kinase domain mutations was also analyzed. Clinical material used in the study consisted of samples from the phase III, adjuvant, FinHer breast cancer trial involving 1010 women. Tumor DNA samples were genotyped for *ERBB4* variants and somatic mutations using matrix-assisted laser desorption ionization/time of flight mass spectrometry. Paraffin-embedded tumor sections from all patients were immunohistochemically stained for ErbB4 expression. Association of *ERBB4* genotype to distant disease-free survival (DDFS) was assessed using Kaplan-Meier and Cox regression analyses. Genotyping was successful for 91–93% of the 1010 samples. Frequencies observed for the *ERBB4* variants were 2.5% and 1.3% for −782G>T and −815A>T, respectively. Variant −815A>T was significantly associated with poor survival (HR  = 2.86 [95% CI 1.15–6.67], *P* = 0.017). In contrast, variant −782G>T was associated with well-differentiated cancer (*P* = 0.019). Two (0.2%) *ERBB4* kinase domain mutations were found, both of which have previously been shown to be functional and promote cancer cell growth *in vitro*. These data present the germ-line *ERBB4* variant −815A>T as a novel prognostic marker in high-risk early breast cancer and indicate the presence of rare but potentially oncogenic somatic *ERBB4* mutations in breast cancer.

## Introduction

Breast cancer is the most frequently diagnosed cancer and the leading cause of death due to a malignancy among women [1]. Hereditary genetic factors are thought to account for approximately 5 to 10% of breast cancers due to germ-line variants in genes that increase the risk for breast cancer, such as *BRCA1* and *BRCA2* [2]. Although a number of germ-line variants have been linked to increased risk of breast cancer [2], less is known about the prognostic significance of hereditary variants.

ErbB4 is a member of the EGF receptor (EGFR) subfamily of receptor tyrosine kinases including EGFR (ErbB1), ErbB2 (HER2, neu), ErbB3 (HER3), and ErbB4 (HER4) [3]. Despite of active research on ErbB4 biology in normal mammary tissue and breast cancer, significance of ErbB4 for breast carcinogenesis is still poorly understood. ErbB4 expression is typically associated with ER- and PR-positivity, ErbB2 receptor-negativity, well-differentiated phenotype and favorable outcome [4]–[7]. On the other hand, ErbB4 overexpression has been associated with shorter relapse-free survival in early, node-negative tumors [8] and with decreased survival in patients with node-positive tumors [9]. Treatment with ErbB4-targeted monoclonal antibody suppresses the growth of breast cancer cells [10], suggesting a possible oncogenic role of ErbB4 in breast cancer.

The role of *ERBB4* gene variation in breast cancer has been less extensively studied. We have previously shown that a single nucleotide polymorphism (SNP) −782 G>T in the promoter region of *ERBB4* gene is a novel risk variant for breast cancer in German population [11]. Two recent studies have also discovered variants of *ERBB4* gene that are associated with increased risk for breast cancer. *ERBB4* SNP rs13393577 was implicated as a new risk variant in a genome-wide association study (GWAS) conducted in Korean population [12], and three *ERBB4* risk variants (rs905883, rs7564590, and rs7558615) were identified in a family-based GWAS in patients of the Framingham heart study [13]. However, no studies have addressed the possible prognostic or predictive value of *ERBB4* variants.

According to the Catalogue Of Somatic Mutations In Cancer (COSMIC), somatic *ERBB4* mutations in breast cancer are rare, as only 1.4% of breast cancers harbor *ERBB4* missense mutations (17 out of 1200 patients) [14]. Although the functional consequences of *ERBB4* breast cancer mutations have not been studied, one *ERBB4* kinase domain mutation (E872K) initially found in breast cancer [15] has later been shown to be functionally active in metastatic melanoma [16].

Here, we analyzed the frequencies and prognostic value of two *ERBB4* promoter variants, −782G>T and −815A>T in a large phase III clinical trial data set of high-risk early breast cancer patients (n = 1010). Frequency of nine specific *ERBB4* kinase domain mutations [15] was also analyzed. The results indicate that the *ERBB4* variant −815A>T was significantly associated with poor distant disease-free survival, indicating for the first time a possible prognostic significance for a genetic variant of *ERBB4* in cancer. The frequency of the analyzed *ERBB4* kinase domain mutations was low (0.2%). However, both somatic mutations had previously been shown to be functional and promote cancer cell growth *in vitro* [16], [17], suggesting a presence of rare but oncogenic *ERBB4* mutations in breast cancer.

## Materials and Methods

### Patient DNA and tumor tissue samples

Study material consisted of DNA and formalin-fixed, paraffin-embedded tissue samples from primary tumors of 1010 women with high-risk early breast cancer who participated in the adjuvant phase III FinHer trial (International Standard Ran- domised Controlled Trial number, ISRCTN76560285) [18]. The key inclusion criteria in the FinHer trial were histologically confirmed invasive breast cancer, age 65 or less, macroscopically complete surgery for breast cancer, presence of at least one positive axillary lymph node or a node-negative breast cancer with tumor diameter at least 20 mm and a negative immunostaining for progesterone steroid hormone receptors. Patients with distant metastases at the time of randomization were excluded. Most (89%) of the study patients had axillary lymph node-positive cancer [18]. All patients were randomly assigned to receive three cycles of vinorelbine or docetaxel together with fluorouracil, epirubicin and cyclophosphamide. Patients with *ERBB2*-positive tumors (n = 232) were also assigned to receive or not to receive adjuvant trastuzumab. The patients signed an informed consent for use of breast tumor tissue samples for research purposes prior to entry to the clinical trial. The protocol of the present study was approved by an Ethics Committee of the Helsinki University Central Hospital.

### Analysis of *ERBB4* variants and somatic mutations

Two *ERBB4* germline single nucleotide variants, −782G>T and −815A>T [11], and nine previously described somatic *ERBB4* mutations [15] were genotyped to establish allele and genotype frequencies in the FinHer cohort. Genotyping was carried out with matrix-assisted laser desorption ionization/time of flight mass spectrometry using SpectroCHIP microarray and Bruker Autoflex (Sequenom) as well as MTP Anchor Chip 400/384 TF and Bruker Ultraflex (Bruker Daltonics) [11]. The *ERBB4* variant analyses were conducted using tumor DNA, as no DNA from non-neoplastic tissue was available. However, when the variants were initially identified from tumor DNA of colorectal cancer patients, the variants were confirmed to be germ-line in all cases [11].

### ErbB4 immunohistochemistry

Paraffin-embedded tumor sections were stained for ErbB4 using HFR-1 monoclonal antibody (2 µg/ml; Abcam), anti-mouse Envision+ System HRP secondary antibody (code K4001; Dako Cytomation), and DAB+ (code K3468; Dako Cytomation) peroxidase substrate. All incubations were carried out in room temperature, and all steps were followed by a rinsing step in 50 mM Tris-HCl pH 7.6 containing 0.05% Tween-20. Sections were counterstained with hematoxylin. ErbB4-positive breast cancer control sections were used as positive controls for each staining series.

### Statistical analyses

Frequency tables were analyzed using the chi-squared test or Fisher's exact test. Survival analyses were carried out with Kaplan-Meier statistics, and survival between groups was compared with the log-rank test. The hazard ratio was computed using a univariable Cox model. Distant disease-free survival was calculated from the date of randomization to the date of detection of distant recurrence of breast cancer or to the date of death whenever death preceded distant recurrence, censoring patients who were alive without distant recurrence on the date of last follow-up [19]. All *P*-values are 2-tailed.

## Results

### Frequencies of *ERBB4* promoter region variants −782G>T and −815A>T

To investigate the prevalence of two *ERBB4* promoter region SNPs, −782G>T and −815A>T, tumor DNA samples from 1010 women with high-risk early breast cancer were analyzed. Successful genotype was obtained from 936 (93%) and 932 (92%) patient samples for *ERBB4* promoter positions −782 and −815, respectively. From these patients, 23 (2.5%) were genotyped to harbor the *ERBB4* −782G>T variant whereas 12 patients (1.3%) harbored the −815A>T variant. All genotypes were heterozygous, with the exception of one homozygous −782TT genotype that was not included in the subsequent statistical analyses.

### Associations of *ERBB4* variants with clinicopathological features and ErbB4 protein expression

When the *ERBB4* promoter region SNP status was compared with clinicopathological characteristics, the −782G>T variant was associated with well-differentiated cancer (*P* = 0.018; Table 1). Neither of the SNPs was significantly associated with primary tumor diameter, axilliary nodal status, histology, tumor grade, ER or PR expression, or *ERBB2* amplification (Table 1). Sixteen (69.6%) out of the 23 cancers with the −782G>T variant were ER-positive and *ERBB2*-negative in immunohistochemical stainings (an approximation for the luminal A biological subtype) as compared with 548 (62.8%) out of the 872 cancers that did not harbor this variant (*P* = 0.510; one of the seven remaining −782G>T cases was ER+/*ERBB2*+, three ER−/*ERBB*2+, and three ER−/*ERBB2*−). Nine (75.0%) of the 12 cancers with the −815A>T variant were ER+ and *ERBB2*− (the remaining three were ER–/*ERBB2*–) as compared with 553 (63.2%) out of the 874 cancers that did not harbor −815A>T (*P* = 0.551). All 8 tumors with the −815A>T variant stained by immunohistochemistry for the p53 protein expression stained negative, whereas 4 (20.0%) out of the 20 cases with the −782G>T variant stained positively for p53 [20].

**Table 1:** Clinicopathological features of patients harboring *ERBB4* variants −782G>T or −815A>T.

	−782 G>T			−815 A>T		
	GG	GT		AA	AT	
	n (%)	n (%)	*P*-value	n (%)	n (%)	*P*-value
						
**Frequency**	872 (98)	23 (2)		879 (99)	12 (1)	
**pT**						
≤20 mm	364 (98)	9 (2)		366 (99)	5 (1)	
>20 mm	508 (98)	13 (2)	0.938	512 (99)	7 (1)	0.999
**pN**						
negative	91 (97)	3 (3)		95 (99)	1 (1)	
positive	781 (98)	20 (2)	0.726	784 (99)	11 (1)	>0.999
**Histology**						
ductal	682 (97)	19 (3)		689 (99)	9 (1)	
lobular/other	190 (98)	4 (2)	0.799	190 (98)	3 (2)	0.729
**Grade**						
1	121 (94)	8 (6)	**0.018**	125 (98)	3 (2)	0.408
2	352 (98)	7 (2)		356 (99)	3 (1)	
3	362 (98)	7 (2)		361 (98)	6 (2)	
**ER**						
-	242 (97)	7 (3)		243 (99)	3 (1)	
+	630 (98)	16 (2)	0.777	636 (99)	9 (1)	>0.999
**PR**						
-	373 (97)	10 (3)		371 (98)	6 (2)	
+	498 (97)	13 (3)	0.959	507 (99)	6 (1)	0.590
***ERBB2***						
-	675 (97)	19 (3)		678 (77)	12 (2)	
+	197 (98)	4 (2)	0.555	201 (23)	0 (0)	0.079
**ErbB4 IHC**						
negative	138 (96)	6 (4)		140 (97)	4 (3)	
positive	697 (98)	15 (2)	0.145	701 (99)	7 (1)	0.098

To address whether the two SNPs regulated ErbB4 expression levels in primary tumors, tumor sections from all the 1010 patients were immunohistochemically stained with a monoclonal antibody recognizing the C-terminus of ErbB4 (HFR-1). However, no associations were found between *ERBB4* SNP status and ErbB4 staining intensity (Table 1). Also, no statistically significant associations were found when cytoplasmic and nuclear ErbB4 staining intensities were separately scored and compared with the *ERBB4* SNP status (data not shown). ErbB4 protein expression did not correlate with patient survival (*P* = 0.826, n = 926), but was strongly associated with ER-positivity (*P* = 0.003) (Supplementary table 1).

### Prognostic significance of *ERBB4* variants

**Figure 1:**
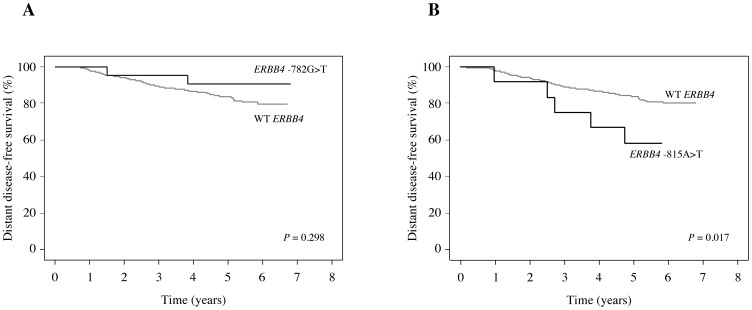
Prognostic associations of the *ERBB4* variants. Kaplan-Meier plots of distant disease-free survival of patients harboring wild-type *ERBB4* or *ERBB4* variants −782G>T (A) or −815A>T (B).

The prognostic significance of the *ERBB4* variants was assessed by analyzing the association of *ERBB4* SNP status with distant disease-free survival (DDFS). No association between *ERBB4* −782G>T and DDFS was found (Figure 1A). In contrast, the *ERBB4* variant −815A>T was significantly associated with poor prognosis (HR = 2.86 [95% CI 1.15-6-67], *P* = 0.017; Figure 1B).

### Frequency of *ERBB4* kinase domain mutations

The frequency of nine previously reported [15] somatic *ERBB4* kinase domain mutations V721I, A773S, R782Q, G802dup, E810K, P854Q, D861Y, E872K, and T926M, including a mutation previously found in breast cancer (E872K), was also analyzed from the tumor DNA samples. The different point mutations were successfully analyzed from 91–93% of the 1010 samples. Two tumors out of all the genotyped tumors were found to harbor *ERBB4* kinase domain mutations G802dup or E872K.

## Discussion

Clinical studies on association of ErbB4 expression with breast cancer patient survival are contradictory [4]–[9], [21], despite *in vitro* as well as *in vivo* mouse xenograft data suggesting an oncogenic role for ErbB4 in breast cancer [10], [22], [23]. However, the prognostic or predictive role of germ-line or somatic *ERBB4* mutations in breast cancer has not been addressed. Here we analyzed the frequencies and prognostic significance of two *ERBB4* genetic variants, −782G>T and −815A>T [11] in a cohort of 1010 patients with high-risk early breast cancer. The frequencies of nine specific *ERBB4* kinase domain mutations [15] was also investigated.

The frequencies of the two *ERBB4* variants were 2.5% (23 out of 936 patients) and 1.3% (12 out of 932 patients) for 782G>T and −815A>T, respectively. In our previous study using samples from German GENICA breast cancer colletion, the frequencies were 5.3% and 1% for 782G>T and −815A>T, respectively [11]. The variant −815A>T was significantly associated with poor prognosis. Interestingly, the variant −782G>T, which was implicated as a risk factor for breast cancer in our previous study [11], was not associated with distant disease-free survival, but with well-differentiated cancer. These data suggest that the heterozygous genotype *ERBB4* −815A/T could be a prognostic marker in high-risk early breast cancer. This is the first indication of prognostic significance for a genetic variant of *ERBB4* in cancer. However, these findings should be confirmed in an independent large patient cohort. The association between the *ERBB4* −815A/T polymorphism and clinical outcome serves the hypothesis that ErbB4-targeted therapy could be beneficial for a subgroup of breast cancer patients in the adjuvant setting.

Immunohistochemical analysis of ErbB4 protein expression levels in the tumors demonstrated that neither of the *ERBB4* variants induced significant changes in ErbB4 expression or subcellular localization in the primary tumors. Total ErbB4 expression also did not associate with DDFS of the patients, but correlated with ER-positivity. This is in accordance with reports associating ErbB4 protein expression with markers of favorable prognosis [4]–[7].

Our analysis of specific *ERBB4* kinase domain mutations revealed two patients harboring somatic ErbB4 mutations G802dup and E872K, respectively. Interestingly, E872K is a mutation initially found in breast cancer [15] that was later also detected in melanoma [16]. The other mutation, G802dup, has previously been reported in non-small cell lung cancer [15]. Both mutations have been shown to be functional and promote cancer cell/tumor growth *in vitro* [16], [17], suggesting the presence of rare but potentially oncogenic *ERBB4* mutations in breast cancer. Although the observed *ERBB4* mutation frequency (0.2%) is low, it corresponds to the frequency of *ERBB2* kinase domain mutations (0.5%) in the same patient cohort [24]. Rare *ERBB2* kinase domain mutations have recently been suggested to serve as predictive markers for ErbB2-targeted therapy in the absence of *ERBB2* amplification [25], indicating that rare mutations may have clinical relevance in high-incidence cancers such as breast cancer.

Taken together, this study presents a genetic *ERBB4* variant as a novel prognostic marker in high-risk early breast cancer and indicates the presence of rare but potentially oncogenic *ERBB4* mutations in breast cancer.

## Supporting Information

Table S1 Associations of immunohistochemical ErbB4 staining intensity with clinicopathological parameters.
(PDF)

## References

[1] Jemal A, Bray F, Ferlay J (2011) Global Cancer Statistics. CA Cancer J Clin 61: 69–90. 21296855 10.3322/caac.20107

[2] Lalloo F, Evans DG (2012) Familial breast cancer. Clin Genet 82: 105–114. 22356477 10.1111/j.1399-0004.2012.01859.x

[3] Hynes NE, MacDonald G (2009) ErbB receptors and signaling pathways in cancer. Curr Opin Cell Biol 21: 177–184. 19208461 10.1016/j.ceb.2008.12.010

[4] Bacus SS, Chin D, Yarden Y, Zelnick CR, Stern DF (1996) Type 1 receptor tyrosine kinases are differentially phosphorylated in mammary carcinoma and differentially associated with steroid receptors. Am J Pathol 148: 549–558. 8579117 PMC1861670

[5] Kew TY, Bell JA, Pinder SE, Denley H, Srinivasan R, et al (2000) c-erbB-4 protein expression in human breast cancer. Br J Cancer 82: 1163–1170. 10735500 10.1054/bjoc.1999.1057 PMC2363344

[6] Sassen A, Rochon J, Wild P, Hartmann A, Hofstaedter F, et al (2008) Cytogenetic analysis of HER1/EGFR, HER2, HER3 and HER4 in 278 breast cancer patients. Breast Cancer Res 10: R2. 18182100 10.1186/bcr1843 PMC2374953

[7] Koutras AK, Kalogeras KT, Dimopoulos M, Wirtz RM, Dafni U, et al.. (2008) Evaluation of the prognostic and predictive value of HER family mRNA expression in high-risk early breast cancer: A Hellenic Cooperative Oncology Group (HeCOG) study: 1775–1785. 10.1038/sj.bjc.6604769 PMC2600696 18985033

[8] Bièche I, Onody P, Tozlu S, Driouch K, Vidaud M, et al (2003) Prognostic value of ERBB family mRNA expression in breast carcinomas. Int J Cancer 106: 758–765. 12866037 10.1002/ijc.11273

[9] Lodge AJ, Anderson JJ, Gullick WJ, Haugk B, Leonard RCF, et al (2003) Type 1 growth factor receptor expression in node positive breast cancer: adverse prognostic significance of c-erbB-4. J Clin Pathol 56: 300–304. 12663644 10.1136/jcp.56.4.300 PMC1769922

[10] Hollmén M, Määttä JA, Bald L, Sliwkowski MX, Elenius K (2009) Suppression of breast cancer cell growth by a monoclonal antibody targeting cleavable ErbB4 isoforms. Oncogene 28: 1309–1319. 19151766 10.1038/onc.2008.481

[11] Rokavec M, Justenhoven C, Schroth W, Istrate MA, Haas S, et al (2007) A novel polymorphism in the promoter region of ERBB4 is associated with breast and colorectal cancer risk. Clin Cancer Res 13: 7506–7514. 18094435 10.1158/1078-0432.CCR-07-0457

[12] Kim H, Lee J-Y, Sung H, Choi J-Y, Park SK, et al (2012) A genome-wide association study identifies a breast cancer risk variant in ERBB4 at 2q34: results from the Seoul Breast Cancer Study. Breast Cancer Res 14: R56. 22452962 10.1186/bcr3158 PMC3446390

[13] Murabito JM, Rosenberg CL, Finger D, Kreger BE, Levy D, et al (2007) A genome-wide association study of breast and prostate cancer in the NHLBI's Framingham Heart Study. BMC Med Genet 8 Suppl 1 S6. 17903305 10.1186/1471-2350-8-S1-S6 PMC1995609

[14] Catalogue of Somatic Mutations In Cancer (COSMIC) website. Available: http://cancer.sanger.ac.uk. Accessed 2014 Jun 23.

[15] Soung YH, Lee JW, Kim SY, Wang YP, Jo KH, et al (2006) Somatic mutations of the ERBB4 kinase domain in human cancers. Int J Cancer 118: 1426–1429. 16187281 10.1002/ijc.21507

[16] Prickett TD, Agrawal NS, Wei X, Yates KE, Lin JC, et al (2009) Analysis of the tyrosine kinome in melanoma reveals recurrent mutations in ERBB4. Nat Genet 41: 1127–1132. 19718025 10.1038/ng.438 PMC2897709

[17] Tvorogov D, Sundvall M, Kurppa K, Hollmén M, Repo S, et al (2009) Somatic mutations of ErbB4: selective loss-of-function phenotype affecting signal transduction pathways in cancer. J Biol Chem 284: 5582–5591. 19098003 10.1074/jbc.M805438200

[18] Joensuu H, Kellokumpu-Lehtinen P-L, Bono P, Alanko T, Kataja V, et al (2006) Adjuvant docetaxel or vinorelbine with or without trastuzumab for breast cancer. N Engl J Med 354: 809–820. 16495393 10.1056/NEJMoa053028

[19] Joensuu H, Bono P, Kataja V, Alanko T, Kokko R, et al (2009) Fluorouracil, epirubicin, and cyclophosphamide with either docetaxel or vinorelbine, with or without trastuzumab, as adjuvant treatments of breast cancer: final results of the FinHer Trial. J Clin Oncol 27: 5685–5692. 19884557 10.1200/JCO.2008.21.4577

[20] Joensuu H, Isola J, Lundin M, Salminen T, Holli K, et al (2003) Amplification of erbB2 and erbB2 Expression Are Superior to Estrogen Receptor Status As Risk Factors for Distant Recurrence in pT1N0M0 Breast Cancer: A Nationwide Population-based Study. Clin Cancer Res 9: 923–930. 12631589

[21] Sundvall M, Iljin K, Kilpinen S, Sara H, Kallioniemi O-P, et al (2008) Role of ErbB4 in breast cancer. J Mammary Gland Biol Neoplasia 13: 259–268. 18454307 10.1007/s10911-008-9079-3

[22] Liu P, Kurppa K, Wildiers H, Reinvall I, Vandorpe T, et al.. (2012) Proteolytic Processing of ErbB4 in Breast Cancer. 7. 10.1371/journal.pone.0039413 PMC3382207 22761786

[23] Muraoka-Cook RS, Sandahl MA, Strunk KE, Miraglia LC, Husted C, et al (2009) ErbB4 splice variants Cyt1 and Cyt2 differ by 16 amino acids and exert opposing effects on the mammary epithelium in vivo. Mol Cell Biol 29: 4935–4948. 19596786 10.1128/MCB.01705-08 PMC2738276

[24] Loi S, Michiels S, Lambrechts D, Fumagalli D, Claes B, et al (2013) Somatic mutation profiling and associations with prognosis and trastuzumab benefit in early breast cancer. J Natl Cancer Inst 105: 960–967. 23739063 10.1093/jnci/djt121 PMC3699437

[25] Bose R, Kavuri SM, Searleman AC, Shen W, Shen D, et al (2013) Activating HER2 mutations in HER2 gene amplification negative breast cancer. Cancer Discov 3: 224–237. 23220880 10.1158/2159-8290.CD-12-0349 PMC3570596

