# Supplementary material for: ERBB4 Promoter Polymorphism Is Associated with Poor Distant Disease-Free Survival in High-Risk Early Breast Cancer
Source: PLoS One. 2014 Jul 18;9(7):e102388. doi: 10.1371/journal.pone.0102388 (PMC4103820; doi:10.1371/journal.pone.0102388)
Supplement: Table S1 — Associations of immunohistochemical ErbB4 staining intensity with clinicopathological parameters. (PDF) [file pone.0102388.s001.pdf]

Supplementary table 1: Associations of immunohistochemical ErbB4 staining intensity with clinicopathological parameters

	ErbB4 staining intensity				
	negative	low	moderate	strong	*P*-value
	n (%)	n (%)	n (%)	n (%)	
**pT**					
<10 mm	13 (18)	28 (40)	25 (35)	5 (7)	
11-20 mm	57 (17)	134 (41)	106 (32)	32 (10)	n = 923
>20 mm	99 (19)	201 (38)	172 (33)	51 (10)	0.976
**pN**					
0	22 (22)	38 (38)	30 (30)	10 (10)	
1-3	100 (18)	229 (41)	180 (32)	54 (9)	n = 926
>3	48 (18)	97 (37)	94 (36)	24 (9)	0.85
**Histology**					
ductal	119 (17)	297 (41)	242 (33)	66 (9)	
lobular	50 (27)	58 (32)	56 (31)	19 (10)	n = 926
other	1 (5)	9 (47)	6 (32)	3 (16)	NA*
**Grade**					
1	27 (20)	53 (40)	42 (32)	10 (8)	
2	65 (18)	140 (38)	122 (33)	41 (11)	n = 887
3	70 (18)	159 (41)	124 (32)	34 (9)	0.844
**ER**					
-	50 (20)	121 (47)	67 (26)	17 (7)	n = 926
+	120 (18)	243 (36)	237 (35)	71 (11)	**0.003**
**PR**					
-	73 (19)	163 (42)	118 (30)	35 (9)	n = 925
+	97 (18)	200 (37)	186 (35)	53 (10)	0.43
ERBB2					
-	136 (19)	275 (38)	248 (34)	61 (9)	n = 926
+	34 (18)	89 (43)	56 (27)	27 (13)	0.052**

* There are too few patients in the "other" category to perform a chin square test. However, there is a difference between ductal and lobular histology; ErbB4 negativity is associated with lobular histology (P = 0.004)

** The nearly significant *P*-value is propably due to coincidence, since there is no clear trend in the percentages
